# Phagocytosis and Inflammation: Exploring the effects of the components of E‐cigarette vapor on macrophages

**DOI:** 10.14814/phy2.13370

**Published:** 2017-09-04

**Authors:** Miranda P. Ween, Jonathan J. Whittall, Rhys Hamon, Paul N. Reynolds, Sandra J. Hodge

**Affiliations:** ^1^ Department of Thoracic Medicine Royal Adelaide Hospital Adelaide Australia; ^2^ School of Medicine University of Adelaide Adelaide Australia

**Keywords:** E‐cigarettes, macrophages, phagocytosis, cytokines, inflammation

## Abstract

E‐cigarettes are perceived as harmless; however, evidence of their safety is lacking. New data suggests E‐cigarettes discharge a range of compounds capable of physiological damage to users. We previously established that cigarette smoke caused defective alveolar macrophage phagocytosis. The present study compared the effect E‐cigarette of components; E‐liquid flavors, nicotine, vegetable glycerine, and propylene glycol on phagocytosis, proinflammatory cytokine secretion, and phagocytic recognition molecule expression using differentiated THP‐1 macrophages. Similar to CSE, phagocytosis of NTHi bacteria was significantly decreased by E‐liquid flavoring (11.65–15.75%) versus control (27.01%). Nicotine also decreased phagocytosis (15.26%). E‐liquid, nicotine, and E‐liquid+ nicotine reduced phagocytic recognition molecules; SR‐A1 and TLR‐2. IL‐8 secretion increased with flavor and nicotine, while TNF *α*, IL‐1*β*, IL‐6, MIP‐1*α*, MIP‐1*β*, and MCP‐1 decreased after exposure to most flavors and nicotine. PG, VG, or PG:VG mix also induced a decrease in MIP‐1*α* and MIP‐1*β*. We conclude that E‐cigarettes can cause macrophage phagocytic dysfunction, expression of phagocytic recognition receptors and cytokine secretion pathways. As such, E‐cigarettes should be treated with caution by users, especially those who are nonsmokers.

## Introduction

Electronic (E)‐cigarettes are a recent innovation, reimagining the cigarette based on designs which have been proposed by inventors since the 60's and finally being commercialized in 2003 in China. They are advertized as a safe way to help people quit smoking tobacco cigarettes, on the basis that only water vapor is inhaled. In recent years, there has been a surge in the number of users, known as ‘vapers’, in many countries. Multiple studies identified most users as those attempting to cease smoking tobacco cigarettes or as an alternative where tobacco cigarette use is prohibited, as summarized in Table 1. However, a growing number of younger people are now using the devices, usually for social and novelty aspects (Schoenborn and Gindi 2015; Walsberger and Havill 2015; Singh et al. 2016; Wilson and Wang 2017). E‐cigarettes are usually filled with an E‐liquid which consists of a base made up of propylene glycol (PG), vegetable glycerine (VG), or a blend of the two, usually with a food safe flavor, and often with nicotine at concentrations varying up to 24 mg/mL. However, up to 250 different compounds have been identified in the resulting, inhaled, E‐liquid vapor (Garcia‐Gomez et al. 2016; Higham et al. 2016), with even more likely given the size cut‐off in these studies. While there is definitive evidence of the dangers of cigarette smoking, the long‐term health risks of E‐cigarettes are not known. The laws governing E‐cigarettes vary across the globe with Australian and Canadian laws being noteworthy by prohibiting the sale of E‐liquid containing nicotine, identifying a need to separate out the effects of the flavoring compounds and nicotine.

**Table 1:** Adult^a^ E‐cigarette use in various countries

Country	Year	Current smoke*r* (%)	Ever users				Current users^a^			
			Total (%)	Nonsmoke*r* (%)	Current smoke*r* (%)	Ex smoke*r* a (%)	Total (%)	Nonsmoke*r* (%)	Current smoke*r* (%)	Ex smoker^a^ (%)
USA^1^	2014		12.6	3.2	47.6	55.4	3.7	0.4	15.9	22.2
Australia (NSW)^2^	2015		8.4	3.2	36.5	N/A	1.3	0.7	4.9	N/A
Canada^3^	2013		8.5	3.0	37.3	5.1	1.8	0.3	9.6	0.9
UK^4^	2012	18.8	5.3	0.5	40.1	3.8	1.6	0.1	13	1.1
Korea^5^	2013	24.1	6.6	0.7	21.8	4.8	1.1	0.2	0.3	4
New Zealand^6^	2014	16.2	13.1	3.4	49. 9	8.4	0.8	0.1	4.0	0.1
Japan^7^	2015	20.5	6.6	3.51	18.3	7.7	1.29	0.6	3.2	1.75

a Definition of “adult”, “current use”, and “ex‐smoker” differs between studies. Ever Use is define as having ever tried an E‐cigarette even once^1‐6^. ^1^Dockrell et al. 2013; ^2^Harrold et al. 2015; ^3^Reid et al. 2015; ^4^Schoenborn and Gindi 2015; ^5^Lee et al. 2016; ^6^Li et al., 2015; ^7^Tabuchi et al. 2016).

Whilst E‐cigarettes are pitched as being a perfectly harmless social pastime, data are emerging that even nonnicotine containing E‐liquids may be damaging to the lungs and airways. E‐liquids have been shown in vitro to: enlarge human lung fibroblast size, induce spindle formation and vacuolization, as well as decrease viability after exposure to individual or mixed components, to a similar degree as cigarette smoke (Lerner et al. 2015). E‐cigarettes cause toxicity to NHBE48 and A549 airway and lung cells via oxidative stress (Cervellati et al. 2014; Scheffler et al. 2015), and both nicotine free and nicotine‐containing E‐cigarette vapor caused loss of lung endothelial barrier function (Schweitzer et al. 2015). In vivo studies in mice show E‐cigarette exposure stunts growth (McGrath‐Morrow et al. 2015) although this may be limited to nicotine‐containing E‐cigarettes (Larcombe et al. 2017). They have also been shown to induce an allergy‐based asthma inflammatory response (Lim and Kim 2014) and increased methacholine response (Larcombe et al. 2017), and increasing susceptibility of mouse lung cells to viral infection (Wu et al. 2014; Sussan et al. 2015). Many of these mouse studies suffer from varied or nonphysiological delivery methods, as well as often lacking a solid basis in in vitro studies, thus this study presents a thorough in vitro study designed to be a basis for future in vivo studies.

We have long been interested in the effects of cigarette smoke on lung cell function. Our identification of defective alveolar macrophage phagocytic function in chronic obstructive disease (COPD) subjects and in response to cigarette smoke (especially with regard to the clearance of apoptotic material, termed efferocytosis), lead to the subsequent discovery of significantly increased apoptosis of epithelial cells in the airway of chronic obstructive pulmonary disease (COPD) subjects and cigarette smokers (Hodge et al. 2005; Hodge et al. 2007; Tran et al. 2016; Ween et al. 2016). These findings implicate defective efferocytosis with secondary necrosis and potentiation of the inflammatory milieu (Hodge et al. 2003, 2005).

In COPD, nontypeable *Haemophilus influenzae* (NTHi) is the most common cause of chronic bacterial airway colonization accounting for up to half of all isolates (King 2012). We found that airway macrophages from cigarette smokers and COPD subjects have a reduced ability to phagocytose bacteria, including NTHi (Hodge et al. 2006; Ween et al. 2016) which can potentially give rise to increased lung infections such as pneumonia. As up to 80% of COPD exacerbations can be attributed to NTHi, clearance by alveolar macrophages is important to reduce bacterial colonization, limit inflammation, and to prevent exacerbations (Sunakawa et al. 2011). Thus, we investigated how E‐cigarettes affect the phagocytic ability of macrophages and explored mechanisms for this effect.

## Materials and Methods

### E‐cigarette and E‐liquids

For all experiments, an EVOD‐2 was used. This device runs at 3.7 V and uses a dual coil with an internal wick and a resistance of 1.5 Ω. Three apple flavors were tested from two suppliers (Aussie Blue and Vape King) in a 70% PG:30% VG base (PG:VG), including one specifically tested to confirm the absence of diacetyl‐acetyl propionyl (E‐liquid 3). Nicotine (in PG from NicVape, SC) at 18 mg/mL in PG alone was also vaporized, also in combination with the three flavors, as well as PG alone (Vape King), VG alone (Vape King), and self mixed PG:VG. Based on the average users puff duration of 2.6 sec (Behar et al. 2015), 50 × 3 sec puffs with 5 sec in between to allow the heating element to cool were bubbled through 10 mL of RPMI 1640 media supplemented with 2 mmol/L l‐glutamine, 10% fetal calf serum (FCS), and Penicillin (12 *μ*g/mL) and Gentamycin (16 *μ*g/mL) (all from Life Technologies, Carlsbad, CA) (culture medium). Control media was obtained, using the same device to pass air through media for the same duration as E‐cigarette use. Cells were treated with 250 *μ*L for 48 well plates and 500 *μ*L for 24 well plates for 24 h.

### Culture and preparation of THP‐1 macrophage cell line

A THP‐1 monocytic cell line (American Type Culture Collection, Manassas, VA) were maintained as per ATCC guidelines in culture medium and 0.05 mmol/L *ß*‐mercaptoethanol. was differentiated into macrophages by seeding at a density of 0.75 × 10^5^ cells in 48 well plastic plates or 1.5 × 10^5^ into 24 well plates, then stimulating with 45 *μ*mol/L phorbol 12‐myristate 13‐acetate (PMA) for 72 h as previously described (Ween et al. 2016). THP‐1 monocytes were differentiated three days prior to treatment. Experiments were carried out within 10 passages and THP‐1 cells were discarded at passage 30 to avoid contamination and genetic mutations from the original source.

### Preparation of NTHI bacteria

A clinical isolate of NTHi was kindly provided by Dr Susan Pizzutto (Menzies School of Health Research, Northern Territory, Australia). NTHi was grown to log phase in Brain Heart Infusion (BHI) broth supplemented with hemin, *β*NAD and glycerol aliquoted and stored at −80°C. Viable quantification (colony‐forming units per mL, cfu/mL) was determined by colony counts of serial dilutions plated onto chocolate agar as described (Ween et al. 2016). In our experience, viability is well maintained at −80°C. Bacteria were permeabilized for 30 min with 70% ethanol, centrifuged at 35,000*g*, washed and resuspended in 1xHBSS 10 mol/L HEPES pH 8.2 and stained with pHrodo Red (2 *μ*g/mL and 2 × 10^6^ cells/mL, Life Technologies) at room temperature for 45 min on a shaker in the dark, washed twice in the HBSS/HEPES solution, resuspended in RPMI, and added at a 100:1 ratio of THP‐1 cells in 500 *μ*L.

### Phagocytosis assay

PMA‐differentiated THP‐1 cells were treated with 250 *μ*L of E‐cigarette media for 24 h. Supernatant was collected, and debris removed by centrifugation at 500*g*. Supernatants were stored at −80°C without protease inhibitor for a max of 6 weeks for LDH assays described below. NTHi were incubated with THP‐1 macrophages for 90 min at 37°C 5% CO_2_. Nonphagocytosed NTHi were removed, the wells rinsed, and the THP‐1 cells incubated with ice cold 1 x HBSS 10 mmol/L HEPES pH 8.2 for 15 min before lifting with a bulb pipette. Macrophages were centrifuged at 1100*g*, vortexed, washed with HBSS/HEPES solution before reading 10,000 events on the FACSCanto II flow cytometer (BD Biosciences, San Jose, CA). As pHrodo is only brightly fluorescent at low pH, found intracellularly, no quenching was required. Macrophages not exposed to NTHi were used as a gating control and phagocytosis was assessed, using FACS DIVA 7.0 (BD Biosciences) and data expressed as the percentage of positive cells. Gating strategies are supplied (Fig. S1).

### Flow cytometry of cell surface markers

Thereafter, 2 × 10^5^ THP‐1 cells were seeded in 24 well plates and differentiated as described above. Cells were treated with E‐cigarette media for 24 h. Supernatant was collected, spun at 500*g* for 10 min to remove debris, and stored at −80°C in the presence of protease inhibitors for cytokine bead array (CBA) analysis as described below. Cells were incubated in ice cold PBS for 15 min before lifting with a bulb pipette. Cells were washed with 0.5% BSA in isoflow (BD Biosciences) and pelleted. Cells were incubated with 2 *μ*L conjugated antibodies (SR‐A1 APC #FAB2708A R& Systems, MN, TLR‐2 PE #FAB6248P, R&D systems, TLR‐4 APC #17‐9917‐41 EBiosciences, CA) for 10 min in the dark, and washed. Unstained‐treated THP‐1 cells were used for gating controls. Fifty thousand events were collected and cell surface markers analyzed, using FACS DIVA 7.0 and expressed as % positive, and also MFI of cells constitutively expressing the marker. Gating strategies are supplied (Fig. S2).

### LDH assay

The Lactate dehydrogenase (LDH) assay was carried out following the manufacturer's instructions (Roche, IN). In a separate well: Tween‐20 was added at 2% v/v, mixed and incubated for 5 min to lyse cells for as a 100% control for total potential max LDH release. Data are presented as a percentage of the lysed cell control for each replicate experiment.

### CSE preparation

Smoke from 2x reference cigarettes (1R5F, University of Kentucky, KY) was bubbled through 10 mL RPMI 1640 media (Life Technologies), supplemented with 12 *μ*g/mL penicillin 16 *μ*g/mL gentamycin (Life Technologies), and l‐glutamine (2 mmol/L, Life Technologies), using a vacuum for 5 min per cigarette. Cigarette smoke extract (CSE) was prepared and used at a concentration of 10%. Cells were treated with 10% CSE or E‐cigarette media for 24 h in parallel experiments.

### Analysis of secreted inflammatory markers via CBA

Cytometric bead arrays (CBA)(BD Biosciences) were performed as per the manufacturer guidelines for cell culture supernatants. CBA were analyzed on a FACSCantoII with FCAP array software. Human soluble protein Flex sets were used, cytokines targeted were TNF*α*, INF*γ*, MIP1*α*, MIP1*β*, IP‐10, IL‐1*β*, IL‐6, IL‐8, IL‐10, IL‐12p70, and MCP‐1.

### Statistical analyses

The Wilcoxon signed rank test for paired data, Kruskal–Wallis nonparametric ANOVA with Mann–Whitney *U* test were employed for statistical analysis. SPSS v23 software was utilized to perform all statistical analysis and differences between groups of *P *< 0.05 considered significant.

## Results

### E‐cigarette components toxicity on THP‐1 macrophages

**Figure 1:**
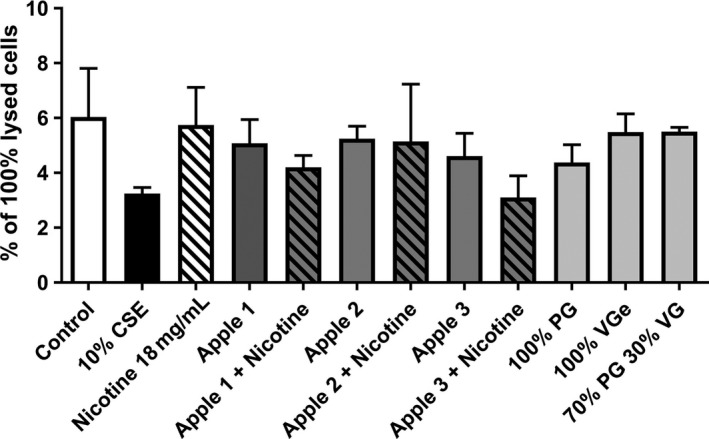
Lactate dehydrogenase (LDH) levels indicating toxicity of E‐cigarette treated cells. THP‐1 PMA‐differentiated macrophages were treated with 10% CSE and media infused with 50 puffs of components of E‐liquids, including three apple flavors ± Nicotine (in 70:30 PG:VG), PG, VG, or 70:30 PG:VG for 24 h. Supernatant was collected, debris removed, and lactate dehydrogenase levels measured and expressed as a percentage of 100% lysis with Tween‐20. *n* = 4 separate experiments. Data represents mean ± SEM.

In this study, we assessed the effects of E‐cigarette vapor on PMA‐differentiated THP‐1 macrophages. Firstly we assessed toxicity of E‐cigarette vapor on THP‐1 macrophages via release of lactate dehydrogenase, and found that none of the three apple flavors, base alone, or nicotine alone induced any significant toxicity at the exposure tested when compared with a total LDH release lysis control (all <6% of lysis max, Fig. 1).

### E‐cigarettes cause decreased macrophage phagocytic capacity

**Figure 2:**
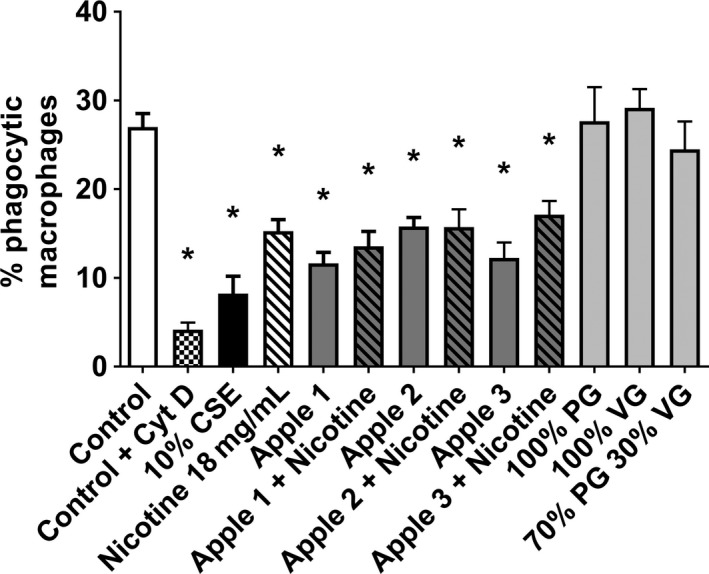
E‐cigarette exposure decreases phagocytic ability of macrophages. THP‐1 PMA‐differentiated macrophages were treated with 10% CSE and media infused with 50 puffs of components of E‐liquids, including three apple flavors ± Nicotine (in 70:30 PG:VG), PG, VG, or 70:30 PG:VG for 24 h. Cells were incubated with pHrodo Red labeled NTHi for 1.5 h, and nonphagocytosed bacteria removed. Due to pH reactivity of pHrodo Red, only THP‐1 with intracellular NTHi were counted as positive in the red channel on a flow cytometer. Expressed as mean percentage positive macrophages ± SEM;* n* = 5 separate experiments performed in duplicate. Significance from control, *P *< 0.05, Mann–Whitney *U* test.

Given that our previous studies had shown decreased alveolar macrophage phagocytic function in COPD subjects and smokers, and in response to cigarette smoke extract in vitro (Hodge et al. 2007), we investigated phagocytosis, using differentiated THP‐1 macrophages exposed to cigarette smoke, and E‐liquid components. Exposure to all three apple flavors caused a significantly decreased ability of macrophages to phagocytose NTHi (11.65–15.79%) versus control (27.01%), as did nicotine at 18 mg/mL (15.26%). There was no statistical difference between E‐liquids with and without nicotine. PG, VG, and PG:VG had no significant effects on phagocytosis (24.5–27.66% vs. 27.01% for control). The reduction in phagocytosis in the presence of 10% CSE (8.2%) was consistent with our previous findings (Tran et al. 2016; Ween et al. 2016) (Fig. 2). The negative control, cytochalasin D, confirmed the assay and gating optimization (Fig. 2).

### E‐cigarettes reduce surface macrophage phagocytosis receptor expression

**Figure 3:**
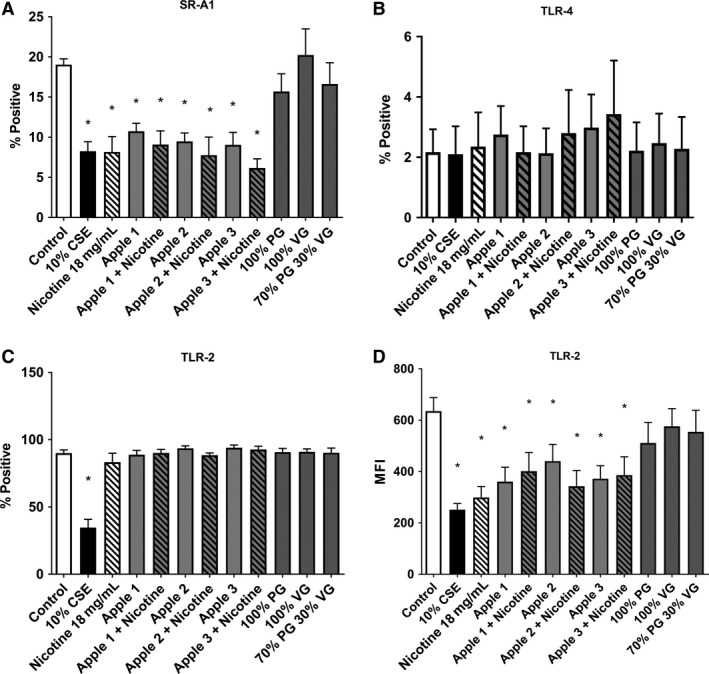
E‐cigarette exposure inhibits phagocytic surface receptor expression. THP‐1 PMA‐differentiated macrophages were treated with 10% CSE and media infused with 50 puffs of the components of E‐liquids, including three apple flavors ± Nicotine (in 70:30 PG:VG), PG, VG, or 70:30 PG:VG for 24 h. Cells were lifted, stained with conjugated antibodies for (A) SR‐A1 (B, C) TLR‐2, and (D) TLR‐4 and percentage positive of MFI measured ± SEM using flow cytometry. *n* = 4 separate experiments performed in duplicate. Significance from control*, P *< 0.05, Mann–Whitney *U* test.

Macrophages express a range of cell surface receptors in order to recognize phagocytic targets including bacteria. Consistent with our previous studies using cigarette smoke, we show that exposure to E‐cigarette vapor also reduces expression of the phagocytosis receptor, scavenger receptor (SR)‐A1 (Fig. 3A). This was also non‐nicotine dependent with all three apple flavors showing a significant decrease in SR‐A1 (9.05–10.75%) versus control treatment (19.04%). Nicotine alone also induced a significant reduction in SR‐A1 (8.18%), whilst PG, VG, and PG:VG showed no significant difference from control treatment. Given the role of toll like receptors (TLR) in the inflammatory response to bacteria, and their known ability to recognize prokaryotic targets, we also investigated the expression of TLR‐2 and TLR‐4. Our data showed that our THP‐1 cells expressed very high levels of TLR‐2 (>90% positive). We found a significant decrease in the percentage of TLR‐2 positive cells with 24 h treatment with 10% CSE (34.77%, Fig. 3B). However, no change in the number of TLR‐2 positive cells when treated with any component of the E‐liquid. Interestingly, when we looked at mean fluorescent intensity (MFI), we found significant decreases when treated with all three flavors (360–440 MFI) as well as nicotine alone (299 MFI) when compared with control (635 MFI), but no significant change with PG, VG, and PG:VG treatment versus control (511–576 MFI, Fig. 3C), indicating a decrease in the number of receptors on each cell. We also observed that THP‐1‐differentiated macrophages had extremely low levels of TLR‐4 expression (<4%, Fig. 3D).

### E‐cigarettes alter cytokine secretion

**Figure 4:**
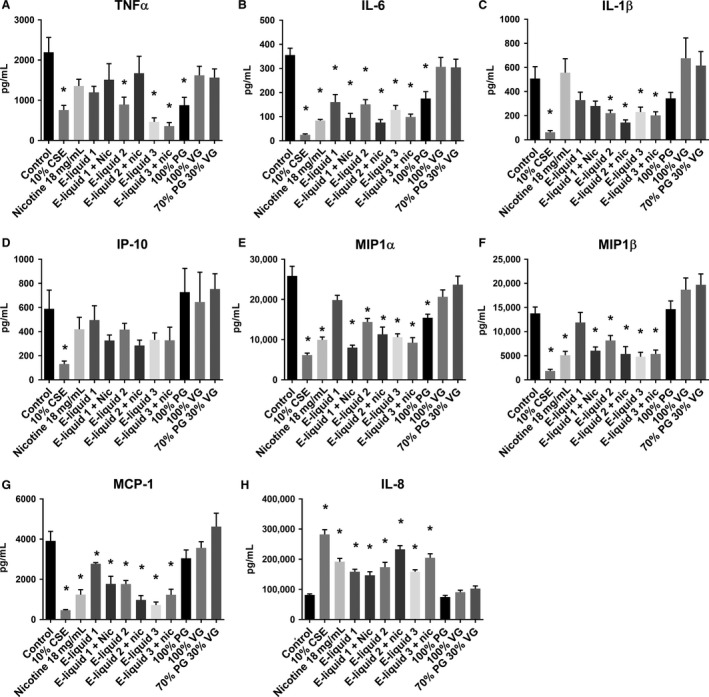
Cytokine profile of E‐cigarette exposed cells. THP‐1 PMA‐differentiated macrophages were treated with 10% CSE and media infused with 50 puffs of the components of E‐liquids, including three apple flavors ± Nicotine (in 70:30 PG:VG), PG, VG, or 70:30 PG:VG for 24 h. Supernatant was collected, debris removed, and (A) IL‐8, (B) TNF *α*, (C) IL‐6, (D) IL‐1*β*, (E) IP‐10, (F) MIP‐1*α*, (G) MIP‐1*β*, and (H) MCP‐1 measured using a cytokine bead array. *n* = 5 experiments assessed in duplicate. Significance from control, *P* < 0.05, Mann–Whitney *U* test. Data represent mean ± SEM.

Cigarette smoking causes significant changes to the inflammatory environment in the lung and alveolar macrophages are a key source of the cytokines involved in the inflammatory process. Thus, we investigated whether E‐cigarette exposure altered the cytokine secretion profile of THP‐1‐differentiated macrophages, using a cytokine bead array that provides very high sensitivity to 5 pg/mL in cell supernatant. We showed that secretion of the neutrophil chemoattractant IL‐8 significantly increased in response to 10% CSE (282526 pg/mL), nicotine (191779 pg/mL), and all three apple flavors (158743–173995 pg/mL), but not to PG, VG, or PG:VG compared with control treatment (81786 pg/mL, Fig. 4A). In contrast, IL‐6, IL‐1*β*, MIP‐1*α*, MIP‐1*β*, MCP‐1, and TNF*α* all significantly decreased in response to 10% CSE as well as E‐liquid 2 and 3, and nicotine. IL‐6 and MCP‐1 also significantly decreased in macrophages treated with E‐liquid 1. Interestingly, MIP‐1*α*, IL‐6, and TNF*α* also showed significant decreases with PG, but not VG or PG:VG. IP‐10 only showed a significant decrease versus control with 10% CSE treatment (Fig. 3B–H). IFN*γ*, IL‐10, and IL‐12p70 were below detection levels.

## Discussion

It has been well documented that tobacco cigarettes are toxic to airway cells (Wu and Lee 1985; Matsumoto et al. 1998; Wright et al. 1999); however, we have described that at lower concentrations, they do not provide a toxic effect to macrophages, but rather affects their function. Whilst E‐cigarettes are a relatively new product on the market, many papers now show that they too have toxic effects on a range of cells (Bahl et al. 2012; McGrath‐Morrow et al. 2015; El Golli et al. 2016; Sancilio et al. 2016; Sherwood and Boitano 2016; Welz et al. 2016) including airway epithelial cells (Scheffler et al. 2015; Schweitzer et al. 2015). This effect is non‐nicotine dependent, and there has been particular interest in certain flavors including tobacco, cherry, and cinnamon, that have been shown to be more toxic than others (Bahl et al. 2012; Behar et al. 2014; Kosmider et al. 2016). In this study, we tested three apple E‐liquids ± added nicotine (18 mg/mL) from two different retailers including one specifically tested to be diacetyl and acetyl propionyl (DA‐AP) free, which has been associated with popcorn lung (Modi et al. 2008; Halldin et al. 2013). We found that there was no cell death associated with the treatment of THP‐1‐differentiated macrophages with these flavors with or without added nicotine (Fig. 1). Our CSE data supported our previous findings (Hodge et al. 2011; Ween et al. 2016) as well as other studies showing cigarette smoke exposure can inhibit bacterial phagocytosis (Shang et al. 2011). To date, there has been no studies on toxicity of E‐cigarettes on macrophages. It is possible that further investigation of a broader range of flavors may identify compounds with toxic effects on macrophages.

NTHi bacteria is the most common lung bacteria in COPD patient exacerbations (King 2012), and it is also common in cystic fibrosis (Bilton et al. 1995) and chronic bronchitis patients (Bandi et al. 2001). We and others have shown that smokers and COPD patient alveolar macrophages (Hodge et al. 2007; Taylor et al. 2010; Berenson et al. 2013) as well as THP‐1‐differentiated macrophages and healthy control alveolar macrophages treated with CSE have decreased ability to phagocytose this bacteria (Bozinovski et al. 2011; Hodge et al. 2011; Thimmulappa et al. 2012; Bain et al. 2016; Ween et al. 2016). Thus, we investigated whether E‐cigarettes caused a similar phagocytic dysfunction utilizing our established THP‐1 model. In this study, we opted to utilize the relatively new dye, pHrodo Red which has minimal fluorescence at neutral to high pH but becomes brightly fluorescent in the low pH environment present in endosomes where phagocytosed bacteria are broken down. This newer stain does not require the quenching of any NTHi bound to the surface of the macrophages that previously used stains required. It also allowed us to differentiate between NTHi bound to the surface of macrophages and those phagocytosed, by placing the cells in pH 3 for 5 min after reading the phagocytosis number. By reading the cells again and comparing the difference in pHrodo red positive cells, we found that very few bacteria were bound to the surface of the macrophages (data not shown). Our data are consistent with other early E‐cigarette studies which show greater survival of MRSA (Hwang et al. 2016) and mouse colonization with *Streptococcus pneumoniae* and influenza viral load (Sussan et al. 2015).

Our laboratory and others had previously shown that one of the reasons why cigarette smoke exposed macrophages had reduced phagocytic ability was a reduced level of expression of a range of bacterial recognition receptors SR‐A1 (Heguy et al. 2006), TLR‐2 (Droemann et al. 2005; Heguy et al. 2006; Kent et al. 2008), and TLR‐4 (Droemann et al. 2005; Heguy et al. 2006; Kent et al. 2008). Thus, we assessed whether exposure to E‐cigarette vapor‐induced reduction of phagocytic ability was similarly linked to bacterial recognition receptors. We observed reduced scavenger receptor SR‐A1 as well as reduced MFI of TLR‐2 on the surface of THP‐1 cells exposed to E‐liquid ± nicotine, and nicotine alone, but not the glycol bases, PG and VG. Some studies have also shown that SR‐A1 deficient mice were more susceptible to pneumococcus and staphylococcus aureus infections (Thomas et al. 2000; Arredouani et al. 2006). This is the first data that shows that E‐cigarettes may alter expression of bacterial recognition receptors on the surface of macrophages resulting in reduced phagocytic ability. We saw very low expression of TLR‐4 on THP‐1 cells, which shows that they are not a 100% ideal model for all bacterial recognition receptors, and further experiments will need to be done with primary alveolar macrophages to investigate the full range of receptors.

We furthermore analyzed the baseline levels of cytokines secreted by THP‐1‐differentiated macrophages exposed to E‐cigarette vapor to determine whether E‐cigarettes could have an effect on inflammatory responses in the lung. We observed an increase in baseline of the neutrophil chemoattractant IL‐8 secretion with exposure to cigarette smoke extract and E‐liquid ± nicotine vapor. IL‐8 secretion has previously been shown to be increased in macrophages exposed to cigarette smoke exposure (Nordskog et al. 2005; Walters et al. 2005; Yang et al. 2007; Birrell et al. 2008; Kent et al. 2008, 2010; Mortaz et al. 2009; Paul‐Clark et al. 2009; Metcalfe et al. 2014) as well as in BALF of smokers (McCrea et al. 1994; Wesselius et al. 1997) versus nonsmokers. However, we saw a decrease in the secretion of a range of other cytokines including TNF*α*, IL‐6, MIP‐1*α*, MIP‐1*β*, MCP‐1, IL‐1*β*, and IP‐10 when treated with E‐liquid ± nicotine, nicotine, or cigarette smoke extract. IL‐12, IFN*γ*, IL10 were undetected in the cells supernatant (Data not shown).

This cigarette smoke result was somewhat surprising at first given previous studies with bronchoalveolar fluid (BALF) and or sputum from smokers and COPD patients had shown increases in some of these cytokines, especially IL‐1*β* (Keatings et al. 1996; Traves et al. 2002; Grumelli et al. 2004; Yang et al. 2007; Gualano et al. 2008; Brozyna et al. 2009; Li et al. 2014). However, further scrutiny of the literature found that many studies had reported similar findings. IL‐6 was decreased in BALF of smokers versus nonsmokers (McCrea et al. 1994) with reduced secretion by cultured alveolar macrophages from smokers compared with nonsmokers (Soliman and Twigg 1992; McCrea et al. 1994; Sauty et al. 1994; Mikuniya et al. 1999; Bardelli et al. 2005; Chen et al. 2007) and those exposed to cigarette smoke (Nordskog et al. 2005; Birrell et al. 2008; Bozinovski et al. 2011). IL‐1*β* secretion also decreased in macrophages exposed to cigarette smoke (Ouyang et al. 2000; Nordskog et al. 2005; Birrell et al. 2008) as well as in cultured alveolar macrophages from smokers compared with nonsmokers (Brown et al. 1989; Soliman and Twigg 1992; Sauty et al. 1994; Birrell et al. 2008). TNF*α* secretion likewise decreased in macrophages exposed to cigarette smoke (Ouyang et al. 2000; Birrell et al. 2008; Bozinovski et al. 2011) and in cultured alveolar macrophages from smokers compared with nonsmokers (Yamaguchi et al. 1993; Sauty et al. 1994; Bardelli et al. 2005; Chen et al. 2007). Similarly, MIP‐1*α* (Birrell et al. 2008), MIP‐1b (Nordskog et al. 2005), IP‐10 (Bozinovski et al. 2011) and MCP‐1 (Nordskog et al. 2005) secretion decreased in macrophages exposed to cigarette smoke.

Limited reports in the literature of the effects of E‐cigarettes and their components on cytokine levels support our findings that E‐cigarettes can induce a cytokine response in cells. IL‐8 was shown to increase in neutrophils exposed to E‐cigarettes (Higham et al. 2016). Decreased IL‐1*β* and TNF‐*α* was observed in PBMCs exposed to nicotine (Ouyang et al. 2000) and further studies showed that cytokines including IL‐6 and MCP‐1 were decreased in the BALF of E‐cigarette exposed mice (Lerner et al. 2015; Sussan et al. 2015). There have been no reports in the literature showing any changes for PG alone (as found in the present study), which we found very interesting given there is great discussion in vaping forums regarding the pros and cons of PG versus VG for taste and vapor production in various E‐cigarette generations, and the awareness that PG has already been shown to be a skin (Lessmann et al. 2005) and lung irritant (Wieslander et al. 2001; Varughese et al. 2005).

The reasons for the decreased cytokine secretion in response to E‐cigarettes and cigarette smoke are unclear; however, the low expression of TLR‐4 on our THP‐1‐differentiated macrophages may have had an effect. IL‐8 is known to be released via a TLR‐2 mediated pathway but not TLR‐4 (Paul‐Clark et al. 2009), while many of the other cytokines are known to be released via a TLR‐4‐mediated pathway (ref). One study showed that MIP‐1*α* and MIP‐1*β* could trigger of TNF‐*α* and IL‐6 secretion by macrophages, so there could also be negative feedback loops in play (O'Grady et al. 1999). Furthermore, TLR‐4‐deficient mice have been shown to have a reduced neutrophil recruitment in response to cigarette smoke as well as a reduced IL‐1*β* and IL‐6 response (Doz et al. 2008). Additionally, this is an in vitro culture system, and alveolar macrophages do not exist in isolation, so it is also likely that cytokine release into BALF is a more complicated procedure involving cross talk between the various cell types present in the airway as well as secretion by more than one cell type. Regardless, we show that E‐cigarettes have an ability to alter cytokine responses from cells, and future studies should look at the BALF of healthy controls compared with vapers to assess physiological cytokine changes in the airways from E‐cigarette use.

We conclude that E‐cigarettes can cause phagocytic dysfunction of macrophages via alteration of bacterial recognition receptors and can alter cytokine secretion pathways. As such, E‐cigarettes should be treated with caution by users, especially those who are nonsmokers, as this data adds to the growing literature showing E‐cigarettes can potentially cause harm to a variety of cells, especially those in the airway where exposure is most direct. Future studies should also focus on whether E‐cigarettes reduce the physiological changes seen in the airways of smokers and any long‐term usage risks.

## Conflict of Interests

The Authors have no competing interests or conflicts of interest to declare. No funding for this study or the authors salaries was received from any tobacco, E‐cigarette, or quitting aid company.

## Data Accessibility

## Supporting information




**Figure S1:** Gating strategy for phrodo phagocytosis assays.

**Figure S2:** Example of gating strategy for marker expression on THP‐1 macrophages.

## References

[phy213370-bib-0001] Arredouani, M. S. , Z. Yang , A. Imrich , Y. Ning , G. Qin , and L. Kobzik . 2006 The macrophage scavenger receptor SR‐AI/II and lung defense against pneumococci and particles. Am. J. Respir. Cell Mol. Biol. 35:474–478. 16675784 10.1165/rcmb.2006-0128OC PMC2643266

[phy213370-bib-0002] Bahl, V. , S. Lin , N. Xu , B. Davis , Y. H. Wang , and P. Talbot . 2012 Comparison of electronic cigarette refill fluid cytotoxicity using embryonic and adult models. Reprod. Toxicol. 34:529–537. 22989551 10.1016/j.reprotox.2012.08.001

[phy213370-bib-0003] Bain, W. G. , A. Tripathi , P. Mandke , J. H. Gans , F. R. D'Alessio , V. K. Sidhaye , et al. 2016 Low‐dose oxygen enhances macrophage‐derived bacterial clearance following cigarette smoke exposure. J. Immunol. Res. 2016:1280347. 27403445 10.1155/2016/1280347 PMC4923598

[phy213370-bib-0004] Bandi, V. , M. A. Apicella , E. Mason , T. F. Murphy , A. Siddiqi , R. L. Atmar , et al. 2001 Nontypeable Haemophilus influenzae in the lower respiratory tract of patients with chronic bronchitis. Am. J. Respir. Crit. Care Med. 164:2114–2119. 11739144 10.1164/ajrccm.164.11.2104093

[phy213370-bib-0005] Bardelli, C. , G. Gunella , F. Varsaldi , P. Balbo , E. Del Boca , I. S. Bernardone , et al. 2005 Expression of functional NK1 receptors in human alveolar macrophages: superoxide anion production, cytokine release and involvement of NF‐kappaB pathway. Br. J. Pharmacol. 145:385–396. 15778738 10.1038/sj.bjp.0706198 PMC1576149

[phy213370-bib-0006] Behar, R. Z. , B. Davis , Y. Wang , V. Bahl , S. Lin , and P. Talbot . 2014 Identification of toxicants in cinnamon‐flavored electronic cigarette refill fluids. Toxicol. In Vitro 28:198–208. 24516877 10.1016/j.tiv.2013.10.006

[phy213370-bib-0007] Behar, R. Z. , M. Hua , and P. Talbot . 2015 Puffing topography and nicotine intake of electronic cigarette users. PLoS ONE 10:e0117222. 25664463 10.1371/journal.pone.0117222 PMC4321841

[phy213370-bib-0008] Berenson, C. S. , R. L. Kruzel , E. Eberhardt , and S. Sethi . 2013 Phagocytic dysfunction of human alveolar macrophages and severity of chronic obstructive pulmonary disease. J. Infect. Dis. 208:2036–2045. 23908477 10.1093/infdis/jit400 PMC3836465

[phy213370-bib-0009] Bilton, D. , A. Pye , M. M. Johnson , J. L. Mitchell , M. Dodd , A. K. Webb , et al. 1995 The isolation and characterization of non‐typeable Haemophilus influenzae from the sputum of adult cystic fibrosis patients. Eur. Respir. J. 8:948–953. 7589381

[phy213370-bib-0010] Birrell, M. A. , S. Wong , M. C. Catley , and M. G. Belvisi . 2008 Impact of tobacco‐smoke on key signaling pathways in the innate immune response in lung macrophages. J. Cell. Physiol. 214:27–37. 17541958 10.1002/jcp.21158

[phy213370-bib-0011] Bozinovski, S. , R. Vlahos , Y. Zhang , L. C. Lah , H. J. Seow , A. Mansell , et al. 2011 Carbonylation caused by cigarette smoke extract is associated with defective macrophage immunity. Am. J. Respir. Cell Mol. Biol. 45:229–236. 20935190 10.1165/rcmb.2010-0272OC

[phy213370-bib-0012] Brown, G. P. , G. K. Iwamoto , M. M. Monick , and G. W. Hunninghake . 1989 Cigarette smoking decreases interleukin 1 release by human alveolar macrophages. Am. J. Physiol. 256:C260–C264. 2784033 10.1152/ajpcell.1989.256.2.C260

[phy213370-bib-0013] Brozyna, S. , J. Ahern , G. Hodge , J. Nairn , M. Holmes , P. N. Reynolds , et al. 2009 Chemotactic mediators of Th1 T‐cell trafficking in smokers and COPD patients. Copd 6:4–16. 19229703 10.1080/15412550902724164

[phy213370-bib-0014] Cervellati, F. , X. M. Muresan , C. Sticozzi , R. Gambari , G. Montagner , H. J. Forman , et al. 2014 Comparative effects between electronic and cigarette smoke in human keratinocytes and epithelial lung cells. Toxicol. In Vitro 28:999–1005. 24809892 10.1016/j.tiv.2014.04.012 PMC4234078

[phy213370-bib-0015] Chen, H. , M. J. Cowan , J. D. Hasday , S. N. Vogel , and A. E. Medvedev . 2007 Tobacco smoking inhibits expression of proinflammatory cytokines and activation of IL‐1R‐associated kinase, p38, and NF‐kappaB in alveolar macrophages stimulated with TLR2 and TLR4 agonists. J. Immunol. 179:6097–6106. 17947684 10.4049/jimmunol.179.9.6097

[phy213370-bib-0016] Dockrell, M. , R. Morrison , L. Bauld , and A. McNeill . 2013 E‐cigarettes: prevalence and attitudes in Great Britain. Nicotine Tob. Res. 15:1737–1744. 23703732 10.1093/ntr/ntt057 PMC3768337

[phy213370-bib-0017] Doz, E. , N. Noulin , E. Boichot , I. Guenon , L. Fick , M. Le Bert , et al. 2008 Cigarette smoke‐induced pulmonary inflammation is TLR4/MyD88 and IL‐1R1/MyD88 signaling dependent. J. Immunol. 180:1169–1178. 18178857 10.4049/jimmunol.180.2.1169

[phy213370-bib-0018] Droemann, D. , T. Goldmann , T. Tiedje , P. Zabel , K. Dalhoff , and B. Schaaf . 2005 Toll‐like receptor 2 expression is decreased on alveolar macrophages in cigarette smokers and COPD patients. Respir. Res. 6:68. 16004610 10.1186/1465-9921-6-68 PMC1187924

[phy213370-bib-0019] El Golli, N. , D. Rahali , A. Jrad‐Lamine , Y. Dallagi , M. Jallouli , Y. Bdiri , et al. 2016 Impact of electronic‐cigarette refill liquid on rat testis. Toxicol. Mech. Methods 26:427–434. 27098213 10.3109/15376516.2016.1163448

[phy213370-bib-0020] Garcia‐Gomez, D. , T. Gaisl , C. Barrios‐Collado , G. Vidal‐de‐Miguel , M. Kohler , and R. Zenobi . 2016 Real‐time chemical analysis of E‐cigarette aerosols by means of secondary electrospray ionization mass spectrometry. Chemistry 22:2452–2457. 26773448 10.1002/chem.201504450

[phy213370-bib-0021] Grumelli, S. , D. B. Corry , L. Z. Song , L. Song , L. Green , J. Huh , et al. 2004 An immune basis for lung parenchymal destruction in chronic obstructive pulmonary disease and emphysema. PLoS Med. 1:e8. 15526056 10.1371/journal.pmed.0010008 PMC523885

[phy213370-bib-0022] Gualano, R. C. , M. J. Hansen , R. Vlahos , J. E. Jones , R. A. Park‐Jones , G. Deliyannis , et al. 2008 Cigarette smoke worsens lung inflammation and impairs resolution of influenza infection in mice. Respir. Res. 9:53. 18627612 10.1186/1465-9921-9-53 PMC2483272

[phy213370-bib-0023] Halldin, C. N. , E. Suarthana , K. B. Fedan , Y. C. Lo , G. Turabelidze , and K. Kreiss . 2013 Increased respiratory disease mortality at a microwave popcorn production facility with worker risk of bronchiolitis obliterans. PLoS ONE 8:e57935. 23469109 10.1371/journal.pone.0057935 PMC3585235

[phy213370-bib-0024] Harrold, T. C. , A. K. Maag , S. Thackway , J. Mitchell , and L. K. Taylor . 2015 Prevalence of e‐cigarette users in New South Wales. Med. J. Aust. 203:326. 26465693 10.5694/mja15.00652

[phy213370-bib-0025] Heguy, A. , T. P. O'Connor , K. Luettich , S. Worgall , A. Cieciuch , B. G. Harvey , et al. 2006 Gene expression profiling of human alveolar macrophages of phenotypically normal smokers and nonsmokers reveals a previously unrecognized subset of genes modulated by cigarette smoking. J. Mol. Med. (Berl) 84:318–328. 16520944 10.1007/s00109-005-0008-2

[phy213370-bib-0026] Higham, A. , N. J. Rattray , J. A. Dewhurst , D. K. Trivedi , S. J. Fowler , R. Goodacre , et al. 2016 Electronic cigarette exposure triggers neutrophil inflammatory responses. Respir. Res. 17:56. 27184092 10.1186/s12931-016-0368-x PMC4869345

[phy213370-bib-0027] Hodge, S. , G. Hodge , R. Scicchitano , P. N. Reynolds , and M. Holmes . 2003 Alveolar macrophages from subjects with chronic obstructive pulmonary disease are deficient in their ability to phagocytose apoptotic airway epithelial cells. Immunol. Cell Biol. 81:289–296. 12848850 10.1046/j.1440-1711.2003.t01-1-01170.x

[phy213370-bib-0028] Hodge, S. , G. Hodge , M. Holmes , and P. N. Reynolds . 2005 Increased airway epithelial and T‐cell apoptosis in COPD remains despite smoking cessation. Eur. Respir. J. 25:447–454. 15738287 10.1183/09031936.05.00077604

[phy213370-bib-0029] Hodge, S. , G. Hodge , S. Brozyna , H. Jersmann , M. Holmes , and P. N. Reynolds . 2006 Azithromycin increases phagocytosis of apoptotic bronchial epithelial cells by alveolar macrophages. Eur. Respir. J. 28:486–495. 16737992 10.1183/09031936.06.00001506

[phy213370-bib-0030] Hodge, S. , G. Hodge , J. Ahern , H. Jersmann , M. Holmes , and P. N. Reynolds . 2007 Smoking alters alveolar macrophage recognition and phagocytic ability: implications in chronic obstructive pulmonary disease. Am. J. Respir. Cell Mol. Biol. 37:748–755. 17630319 10.1165/rcmb.2007-0025OC

[phy213370-bib-0031] Hodge, S. , G. Matthews , V. Mukaro , J. Ahern , A. Shivam , G. Hodge , et al. 2011 Cigarette smoke‐induced changes to alveolar macrophage phenotype and function are improved by treatment with procysteine. Am. J. Respir. Cell Mol. Biol. 44:673–681. 20595463 10.1165/rcmb.2009-0459OC

[phy213370-bib-0032] Hwang, J. H. , M. Lyes , K. Sladewski , S. Enany , E. McEachern , D. P. Mathew , et al. 2016 Electronic cigarette inhalation alters innate immunity and airway cytokines while increasing the virulence of colonizing bacteria. J. Mol. Med. (Berl) 94:667–679. 26804311 10.1007/s00109-016-1378-3

[phy213370-bib-0033] Keatings, V. M. , P. D. Collins , D. M. Scott , and P. J. Barnes . 1996 Differences in interleukin‐8 and tumor necrosis factor‐alpha in induced sputum from patients with chronic obstructive pulmonary disease or asthma. Am. J. Respir. Crit. Care Med. 153:530–534. 8564092 10.1164/ajrccm.153.2.8564092

[phy213370-bib-0034] Kent, L. , L. Smyth , C. Clayton , L. Scott , T. Cook , R. Stephens , et al. 2008 Cigarette smoke extract induced cytokine and chemokine gene expression changes in COPD macrophages. Cytokine 42:205–216. 18358739 10.1016/j.cyto.2008.02.001

[phy213370-bib-0035] Kent, L. M. , S. M. Fox , S. N. Farrow , and D. Singh . 2010 The effects of dexamethasone on cigarette smoke induced gene expression changes in COPD macrophages. Int. Immunopharmacol. 10:57–64. 19818417 10.1016/j.intimp.2009.09.021

[phy213370-bib-0036] King, P. 2012 Haemophilus influenzae and the lung (Haemophilus and the lung). Clin. Trans. Med. 1:10. 10.1186/2001-1326-1-10 PMC3567431 23369277

[phy213370-bib-0037] Kosmider, L. , A. Sobczak , A. Prokopowicz , J. Kurek , M. Zaciera , J. Knysak , et al. 2016 Cherry‐flavoured electronic cigarettes expose users to the inhalation irritant, benzaldehyde. Thorax 71:376–377. 26822067 10.1136/thoraxjnl-2015-207895 PMC4937616

[phy213370-bib-0038] Larcombe, A. N. , M. A. Janka , B. J. Mullins , L. J. Berry , A. Bredin , and P. J. Franklin . 2017 The effects of electronic cigarette aerosol exposure on inflammation and lung function in mice. Am. J. Physiol. Lung Cell. Mol. Physiol. 00203:02016. 10.1152/ajplung.00203.2016 28360111

[phy213370-bib-0039] Lee, J. A. , S. H. Kim , and H. J. Cho . 2016 Electronic cigarette use among Korean adults. Int J Public Health 61:151–157. 26564162 10.1007/s00038-015-0763-y

[phy213370-bib-0040] Lerner, C. A. , I. K. Sundar , H. Yao , J. Gerloff , D. J. Ossip , S. McIntosh , et al. 2015 Vapors produced by electronic cigarettes and e‐juices with flavorings induce toxicity, oxidative stress, and inflammatory response in lung epithelial cells and in mouse lung. PLoS ONE 10:e0116732. 25658421 10.1371/journal.pone.0116732 PMC4319729

[phy213370-bib-0041] Lessmann, H. , A. Schnuch , J. Geier , and W. Uter . 2005 Skin‐sensitizing and irritant properties of propylene glycol. Contact Derm. 53:247–259. 16283903 10.1111/j.0105-1873.2005.00693.x

[phy213370-bib-0500] Li, J. , R. Newcombe , and D. Walton . 2015 The prevalence, correlates and reasons for using electronic cigarettes among New Zealand adults. Addict Behav. 45:245–351. 25744712 10.1016/j.addbeh.2015.02.006

[phy213370-bib-0042] Li, J. R. , W. X. Zhou , Z. X. Zhao , and J. M. Gao . 2014 Differential expression of the inflammation‐associated chemokines/cytokines in mouse lung after exposure to cigarette smoke and smoking cessation. Zhongguo Yi Xue Ke Xue Yuan Xue Bao 36:241–248. 24997814 10.3881/j.issn.1000-503X.2014.03.003

[phy213370-bib-0043] Lim, H. B. , and S. H. Kim . 2014 Inhallation of e‐cigarette cartridge solution aggravates allergen‐induced airway inflammation and hyper‐responsiveness in mice. Toxicol. Res. 30:13–18. 24795794 10.5487/TR.2014.30.1.013 PMC4007038

[phy213370-bib-0044] Matsumoto, K. , H. Aizawa , H. Inoue , H. Koto , S. Takata , M. Shigyo , et al. 1998 Eosinophilic airway inflammation induced by repeated exposure to cigarette smoke. Eur. Respir. J. 12:387–394. 9727790 10.1183/09031936.98.12020387

[phy213370-bib-0045] McCrea, K. A. , J. E. Ensor , K. Nall , E. R. Bleecker , and J. D. Hasday . 1994 Altered cytokine regulation in the lungs of cigarette smokers. Am. J. Respir. Crit. Care Med. 150:696–703. 8087340 10.1164/ajrccm.150.3.8087340

[phy213370-bib-0046] McGrath‐Morrow, S. A. , M. Hayashi , A. Aherrera , A. Lopez , A. Malinina , J. M. Collaco , et al. 2015 The effects of electronic cigarette emissions on systemic cotinine levels, weight and postnatal lung growth in neonatal mice. PLoS ONE 10:e0118344. 25706869 10.1371/journal.pone.0118344 PMC4338219

[phy213370-bib-0047] Metcalfe, H. J. , S. Lea , D. Hughes , R. Khalaf , K. Abbott‐Banner , and D. Singh . 2014 Effects of cigarette smoke on Toll‐like receptor (TLR) activation of chronic obstructive pulmonary disease (COPD) macrophages. Clin. Exp. Immunol. 176:461–472. 24528166 10.1111/cei.12289 PMC4008991

[phy213370-bib-0048] Mikuniya, T. , S. Nagai , T. Tsutsumi , K. Morita , T. Mio , N. Satake , et al. 1999 Proinflammatory or regulatory cytokines released from BALF macrophages of healthy smokers. Respiration 66:419–426. 10516538 10.1159/000029425

[phy213370-bib-0049] Modi, P. , V. Yadava , R. Sreedhar , F. Khasawaneh , and R. A. Balk . 2008 A case of flavor‐induced lung disease. South. Med. J. 101:541–542. 18414158 10.1097/SMJ.0b013e31816bead7

[phy213370-bib-0050] Mortaz, E. , Z. Lazar , L. Koenderman , A. D. Kraneveld , F. P. Nijkamp , and G. Folkerts . 2009 Cigarette smoke attenuates the production of cytokines by human plasmacytoid dendritic cells and enhances the release of IL‐8 in response to TLR‐9 stimulation. Respir. Res. 10:47. 19515231 10.1186/1465-9921-10-47 PMC2701931

[phy213370-bib-0051] Nordskog, B. K. , W. R. Fields , and G. M. Hellmann . 2005 Kinetic analysis of cytokine response to cigarette smoke condensate by human endothelial and monocytic cells. Toxicology 212:87–97. 15885868 10.1016/j.tox.2005.04.005

[phy213370-bib-0052] O'Grady, N. P. , M. Tropea , H. L. 2nd Preas , D. Reda , R. W. Vandivier , S. M. Banks , et al. 1999 Detection of macrophage inflammatory protein (MIP)‐1alpha and MIP‐1beta during experimental endotoxemia and human sepsis. J. Infect. Dis. 179:136–141. 9841832 10.1086/314559

[phy213370-bib-0053] Ouyang, Y. , N. Virasch , P. Hao , M. T. Aubrey , N. Mukerjee , B. E. Bierer , et al. 2000 Suppression of human IL‐1beta, IL‐2, IFN‐gamma, and TNF‐alpha production by cigarette smoke extracts. J. Allergy Clin. Immunol. 106:280–287. 10932071 10.1067/mai.2000.107751

[phy213370-bib-0054] Paul‐Clark, M. J. , S. K. McMaster , R. Sorrentino , S. Sriskandan , L. K. Bailey , L. Moreno , et al. 2009 Toll‐like receptor 2 is essential for the sensing of oxidants during inflammation. Am. J. Respir. Crit. Care Med. 179:299–306. 19011150 10.1164/rccm.200707-1019OC PMC2643079

[phy213370-bib-0055] Reid, J. L. , V. L. Rynard , C. D. Czoli , and D. Hammond . 2015 Who is using e‐cigarettes in Canada? Nationally representative data on the prevalence of e‐cigarette use among Canadians. Prev. Med. 81:180–183. 26348453 10.1016/j.ypmed.2015.08.019

[phy213370-bib-0056] Sancilio, S. , M. Gallorini , A. Cataldi , and V. di Giacomo . 2016 Cytotoxicity and apoptosis induction by e‐cigarette fluids in human gingival fibroblasts. Clin. Oral Investig. 20:477–483. 10.1007/s00784-015-1537-x 26239821

[phy213370-bib-0057] Sauty, A. , J. Mauel , M. M. Philippeaux , and P. Leuenberger . 1994 Cytostatic activity of alveolar macrophages from smokers and nonsmokers: role of interleukin‐1 beta, interleukin‐6, and tumor necrosis factor‐alpha. Am. J. Respir. Cell Mol. Biol. 11:631–637. 7946392 10.1165/ajrcmb.11.5.7946392

[phy213370-bib-0058] Scheffler, S. , H. Dieken , O. Krischenowski , C. Forster , D. Branscheid , and M. Aufderheide . 2015 Evaluation of E‐cigarette liquid vapor and mainstream cigarette smoke after direct exposure of primary human bronchial epithelial cells. Int. J. Environ. Res. Public Health 12:3915–3925. 25856554 10.3390/ijerph120403915 PMC4410224

[phy213370-bib-0059] Schoenborn, C. A. , and R. M. Gindi . 2015 Electronic cigarette use among adults: United States, 2014. NCHS Data Brief 000: 1–8. 26555932

[phy213370-bib-0060] Schweitzer, K. S. , S. X. Chen , S. Law , M. J. Van Demark , C. Poirier , M. J. Justice , et al. 2015 Endothelial disruptive pro‐inflammatory effects of nicotine and e‐cigarette vapor exposures. Am. J. Physiol. Lung Cell. Mol. Physiol. 00411:02015. 10.1152/ajplung.00411.2014 PMC4504977 25979079

[phy213370-bib-0061] Shang, S. , D. Ordway , M. Henao‐Tamayo , X. Bai , R. Oberley‐Deegan , C. Shanley , et al. 2011 Cigarette smoke increases susceptibility to tuberculosis–evidence from in vivo and in vitro models. J. Infect. Dis. 203:1240–1248. 21357942 10.1093/infdis/jir009

[phy213370-bib-0062] Sherwood, C. L. , and S. Boitano . 2016 Airway epithelial cell exposure to distinct e‐cigarette liquid flavorings reveals toxicity thresholds and activation of CFTR by the chocolate flavoring 2,5‐dimethypyrazine. Respir. Res. 17:57. 27184162 10.1186/s12931-016-0369-9 PMC4869201

[phy213370-bib-0063] Singh, T. , S. Kennedy , K. Marynak , A. Persoskie , P. Melstrom , and B. A. King . 2016 Characteristics of electronic cigarette use among middle and high school students ‐ United States, 2015. MMWR Morb. Mortal. Wkly Rep. 65:1425–1429. 28033310 10.15585/mmwr.mm655051a2

[phy213370-bib-0064] Soliman, D. M. , and H. L. 3rd Twigg . 1992 Cigarette smoking decreases bioactive interleukin‐6 secretion by alveolar macrophages. Am. J. Physiol. 263:L471–L478. 1415725 10.1152/ajplung.1992.263.4.L471

[phy213370-bib-0065] Sunakawa, K. , Y. Takeuchi , and S. Iwata . 2011 Nontypeable haemophilus influenzae (NTHi) epidemiology. Kansenshogaku Zasshi 85:227–237. 21706841 10.11150/kansenshogakuzasshi.85.227

[phy213370-bib-0066] Sussan, T. E. , S. Gajghate , R. K. Thimmulappa , J. Ma , J. H. Kim , K. Sudini , et al. 2015 Exposure to electronic cigarettes impairs pulmonary anti‐bacterial and anti‐viral defenses in a mouse model. PLoS ONE 10:e0116861. 25651083 10.1371/journal.pone.0116861 PMC4317176

[phy213370-bib-0067] Tabuchi, T. , K. Kiyohara , T. Hoshino , K. Bekki , Y. Inaba , and N. Kunugita . 2016 Awareness and use of electronic cigarettes and heat‐not‐burn tobacco products in Japan. Addiction 111:706–713. 26566956 10.1111/add.13231

[phy213370-bib-0068] Taylor, A. E. , T. K. Finney‐Hayward , J. K. Quint , C. M. Thomas , S. J. Tudhope , J. A. Wedzicha , et al. 2010 Defective macrophage phagocytosis of bacteria in COPD. Eur. Respir. J. 35:1039–1047. 19897561 10.1183/09031936.00036709

[phy213370-bib-0069] Thimmulappa, R. K. , X. Gang , J. H. Kim , T. E. Sussan , J. L. Witztum , and S. Biswal . 2012 Oxidized phospholipids impair pulmonary antibacterial defenses: evidence in mice exposed to cigarette smoke. Biochem. Biophys. Res. Commun. 426:253–259. 22935414 10.1016/j.bbrc.2012.08.076 PMC3495329

[phy213370-bib-0070] Thomas, C. A. , Y. Li , T. Kodama , H. Suzuki , S. C. Silverstein , and J. El Khoury . 2000 Protection from lethal gram‐positive infection by macrophage scavenger receptor‐dependent phagocytosis. J. Exp. Med. 191:147–156. 10620613 10.1084/jem.191.1.147 PMC2195800

[phy213370-bib-0071] Tran, H. B. , J. Barnawi , M. Ween , R. Hamon , E. Roscioli , G. Hodge , et al. 2016 Cigarette smoke inhibits efferocytosis via deregulation of sphingosine kinase signaling: reversal with exogenous S1P and the S1P analogue FTY720. J. Leukoc. Biol. 100:195–202. 26792820 10.1189/jlb.3A1015-471R

[phy213370-bib-0072] Traves, S. L. , S. V. Culpitt , R. E. Russell , P. J. Barnes , and L. E. Donnelly . 2002 Increased levels of the chemokines GROalpha and MCP‐1 in sputum samples from patients with COPD. Thorax 57:590–595. 12096201 10.1136/thorax.57.7.590 PMC1746378

[phy213370-bib-0073] Varughese, S. , K. Teschke , M. Brauer , Y. Chow , C. van Netten , and S. M. Kennedy . 2005 Effects of theatrical smokes and fogs on respiratory health in the entertainment industry. Am. J. Ind. Med. 47:411–418. 15828073 10.1002/ajim.20151

[phy213370-bib-0074] Walsberger, S. , and M. Havill . 2015 NSW community behaviors, beliefs & attitudes towards E‐cigarettes: results of an online survey cancer council. NSW, Sydney.

[phy213370-bib-0075] Walters, M. J. , M. J. Paul‐Clark , S. K. McMaster , K. Ito , I. M. Adcock , and J. A. Mitchell . 2005 Cigarette smoke activates human monocytes by an oxidant‐AP‐1 signaling pathway: implications for steroid resistance. Mol. Pharmacol. 68:1343–1353. 16061772 10.1124/mol.105.012591

[phy213370-bib-0076] Ween, M. , J. Ahern , A. Carroll , G. Hodge , S. Pizzutto , H. Jersmann , et al. 2016 A small volume technique to examine and compare alveolar macrophage phagocytosis of apoptotic cells and non typeable Haemophilus influenzae (NTHi). J. Immunol. Methods 429:7–14. 26678160 10.1016/j.jim.2015.12.004

[phy213370-bib-0077] Welz, C. , M. Canis , S. Schwenk‐Zieger , S. Becker , V. Stucke , F. Ihler , et al. 2016 Cytotoxic and genotoxic effects of electronic cigarette liquids on human mucosal tissue cultures of the oropharynx. J. Environ. Pathol. Toxicol. Oncol. 35:343–354. 27992314 10.1615/JEnvironPatholToxicolOncol.2016016652

[phy213370-bib-0078] Wesselius, L. J. , M. E. Nelson , K. Bailey , and A. R. O'Brien‐Ladner . 1997 Rapid lung cytokine accumulation and neutrophil recruitment after lipopolysaccharide inhalation by cigarette smokers and nonsmokers. J. Lab. Clin. Med. 129:106–114. 9011586 10.1016/s0022-2143(97)90167-0

[phy213370-bib-0079] Wieslander, G. , D. Norback , and T. Lindgren . 2001 Experimental exposure to propylene glycol mist in aviation emergency training: acute ocular and respiratory effects. Occup. Environ. Med. 58:649–655. 11555686 10.1136/oem.58.10.649 PMC1740047

[phy213370-bib-0080] Wilson, F. A. , and Y. Wang . 2017 Recent findings on the prevalence of E‐cigarette use among adults in the U.S. Am. J. Prev. Med. 52:385–390. 27988090 10.1016/j.amepre.2016.10.029

[phy213370-bib-0081] Wright, J. L. , J. P. Sun , and A. Churg . 1999 Cigarette smoke exposure causes constriction of rat lung. Eur. Respir. J. 14:1095–1099. 10596696 10.1183/09031936.99.14510959

[phy213370-bib-0082] Wu, Z. X. , and L. Y. Lee . 1985 Airway hyperresponsiveness induced by chronic exposure to cigarette smoke in guinea pigs: role of tachykinins. J. Appl. Physiol. 87(1621–1628):1999. 10.1152/jappl.1999.87.5.1621 10562600

[phy213370-bib-0083] Wu, Q. , D. Jiang , M. Minor , and H. W. Chu . 2014 Electronic cigarette liquid increases inflammation and virus infection in primary human airway epithelial cells. PLoS ONE 9:e108342. 25244293 10.1371/journal.pone.0108342 PMC4171526

[phy213370-bib-0084] Yamaguchi, E. , A. Itoh , K. Furuya , H. Miyamoto , S. Abe , and Y. Kawakami . 1993 Release of tumor necrosis factor‐alpha from human alveolar macrophages is decreased in smokers. Chest 103:479–483. 8432140 10.1378/chest.103.2.479

[phy213370-bib-0085] Yang, S. R. , J. Wright , M. Bauter , K. Seweryniak , A. Kode , and I. Rahman . 2007 Sirtuin regulates cigarette smoke‐induced proinflammatory mediator release via RelA/p65 NF‐kappaB in macrophages in vitro and in rat lungs in vivo: implications for chronic inflammation and aging. Am. J. Physiol. Lung Cell. Mol. Physiol. 292:L567–L576. 17041012 10.1152/ajplung.00308.2006

