# Supplementary figures and images for: Phagocytosis and Inflammation: Exploring the effects of the components of E‐cigarette vapor on macrophages
Source: Physiol Rep. 2017 Sep 4;5(16):e13370. doi: 10.14814/phy2.13370 (PMC5582261; doi:10.14814/phy2.13370)

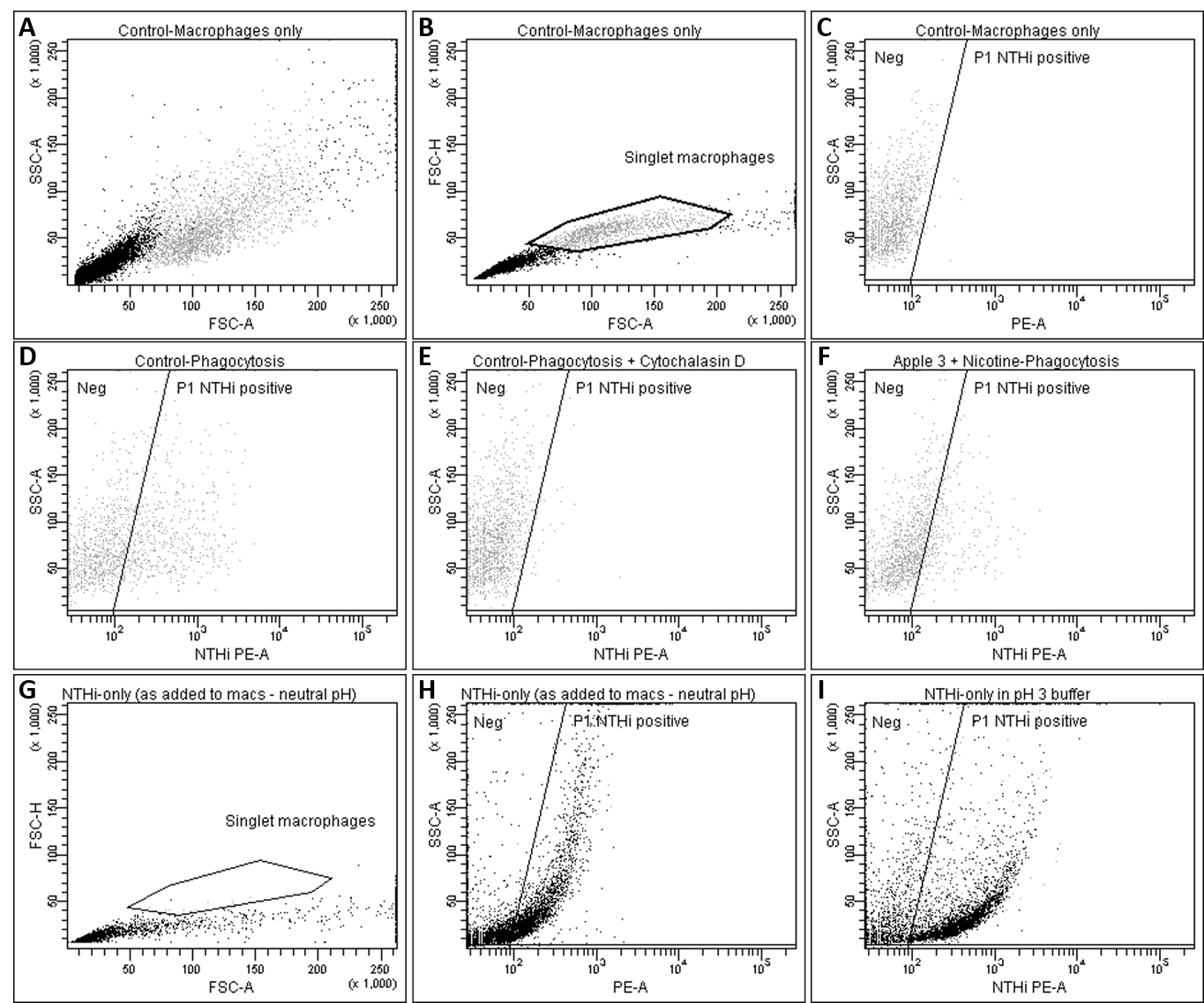

Supplement: Supplementary file 1 — Figure S1: Gating strategy for phrodo phagocytosis assays. [file PHY2-5-e13370-s001.tif]

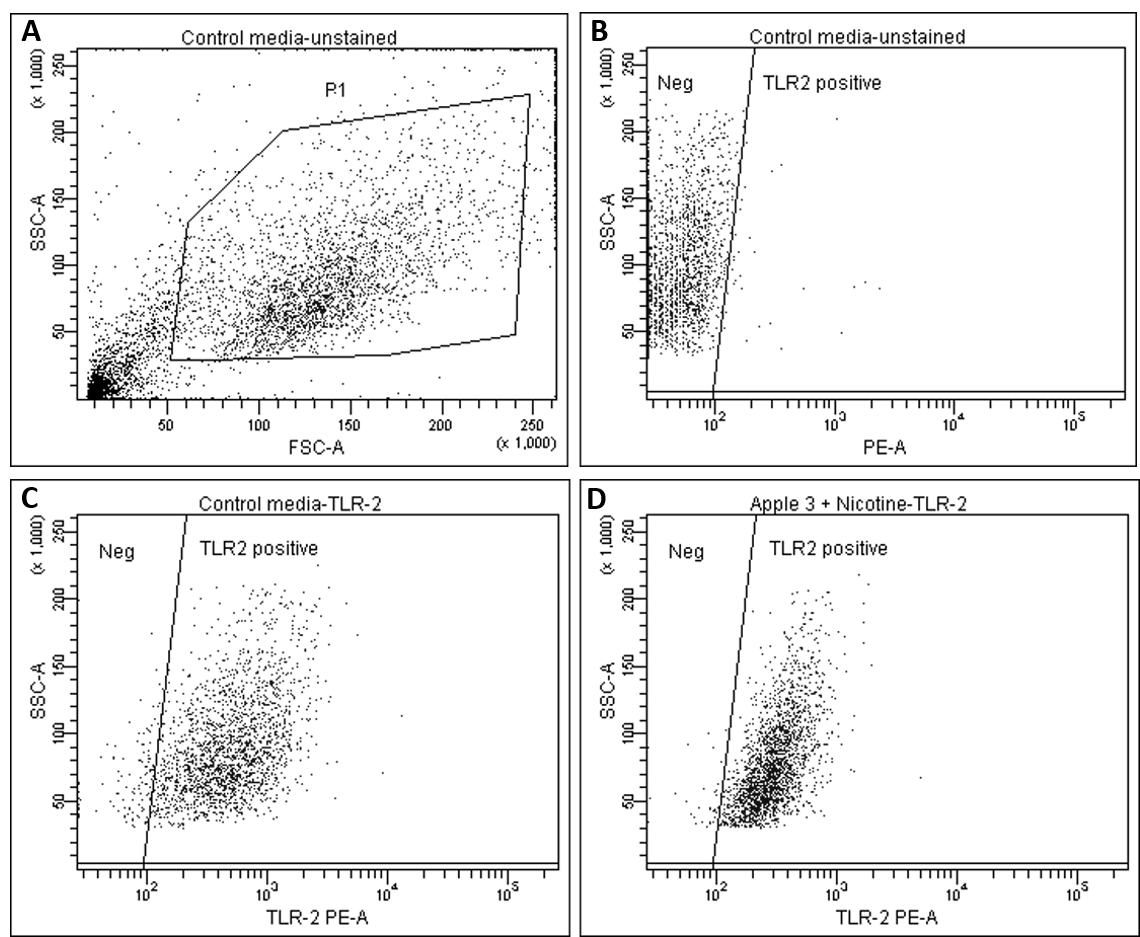

Supplement: Supplementary file 2 — Figure S2: Example of gating strategy for marker expression on THP‐1 macrophages. [file PHY2-5-e13370-s002.tif]
