# Supplementary material for: Phagocytosis and Inflammation: Exploring the effects of the components of E‐cigarette vapor on macrophages
Source: Physiol Rep. 2017 Sep 4;5(16):e13370. doi: 10.14814/phy2.13370 (PMC5582261; doi:10.14814/phy2.13370)
Supplement: Supplementary file 3 [file PHY2-5-e13370-s003.docx]

**Figure S1. Gating Strategy for pHrodo Phagocytosis Assays**

(A) Forward and Side scatter of macrophages (B) Selection of singlet macrophages (C) Macrophage only sample to show negative/positive cut-off (D) Control treated macrophages exposed to pHrodo labelled NTHi showing macrophages positive for internal NTHi (E) Control macrophages exposed to pHrodo labelled NTHi also treated with phagocytosis inhibitor, Cytochalasin D (F) Macrophages treated with E-cigarette vapour infused media containing nicotine exposed to pHrodo labelled NTHi (G) evidence that pHrodo labelled NTHi are excluded by selective size gating for macrophages (H) and (I) evidence that the pHrodo labelled NTHi increase their fluorescence when exposed to low pH.

**Figure S2. Example of gating strategy for marker expression on THP-1 macrophages**

(A) Selection of macrophages based on forward and side scatter (B) unstained control treated macrophages to show negative/positive cut offs (C) TLR-2 positive control treated macrophages (D) Macrophages treated with E-cigarette vapour infused media containing nicotine showing reduced MFI for TLR-2 staining
